# Electrophysiological evidence for notation independence in numerical processing

**DOI:** 10.1186/1744-9081-3-1

**Published:** 2007-01-10

**Authors:** Melissa E Libertus, Marty G Woldorff, Elizabeth M Brannon

**Affiliations:** 1 Center for Cognitive Neuroscience, Box 90999, Duke University, NC 27708, USA; 2 Department of Psychology and Neuroscience, Box 90086, Duke University, NC 27708, USA; 3 Department of Psychiatry, Duke University, NC 27708, USA

## Abstract

**Background:**

A dominant view in numerical cognition is that numerical comparisons operate on a notation independent representation (Dehaene, 1992). Although previous human neurophysiological studies using scalp-recorded event-related potentials (ERPs) on the numerical distance effect have been interpreted as supporting this idea, differences in the electrophysiological correlates of the numerical distance effect in symbolic notations (e.g. Arabic numerals) and non-symbolic notations (e.g. a set of visually presented dots of a certain number) are not entirely consistent with this view.

**Methods and results:**

Two experiments were conducted to resolve these discrepancies. In Experiment 1, participants performed a symbolic and a non-symbolic numerical comparison task ("smaller or larger than 5?") with numerical values 1–4 and 6–9 while ERPs were recorded. Consistent with a previous report (Temple & Posner, 1998), in the symbolic condition the amplitude of the P2p ERP component (210–250 ms post-stimulus) was larger for values near to the standard than for values far from the standard whereas this pattern was reversed in the non-symbolic condition. However, closer analysis indicated that the reversal in polarity was likely due to the presence of a confounding stimulus effect on the early sensory ERP components for small versus larger numerical values in the non-symbolic condition. In Experiment 2 exclusively large numerosities (8–30) were used, thereby rendering sensory differences negligible, and with this control in place the numerical distance effect in the non-symbolic condition mirrored the symbolic condition of Experiment 1.

**Conclusion:**

Collectively, the results support the claim of an abstract semantic processing stage for numerical comparisons that is independent of input notation.

## Background

There are many everyday situations in which we need to compare the number of items in two or more sets. For instance, when shopping one must compare the funds one possesses to the cost of the bill to determine if the purchase can be made. Or when choosing the most efficient line for checkout at the grocery store, one typically compares estimates of the number of customers in each line. Many such comparisons are seemingly performed effortlessly, and humans as well as non-human primates and other animals estimate and compare the numerosity of both visual and auditory sets (see [1] for a review).

In a seminal study, Moyer and Landauer [2] implemented these everyday skills in a laboratory setting. Adults were presented with pairs of Arabic numerals between 1 and 9 and were required to choose the smaller or larger value. Accuracy and response time (RT) were systematically affected by the numerical disparity between the quantities represented by the Arabic numerals. The larger the numerical disparity, the faster and more accurate were the judgments, a phenomenon known as the distance effect. In addition, when distance was held constant, accuracy decreased and RT increased for larger magnitudes, a phenomenon known as the size effect. The distance and size effect together yield ratio dependent discrimination that follows Weber's law. Weber's law is well-known to describe discrimination for continuous quantities such as weight [3] or object size [4], and the fact that it applies to number discrimination suggests – despite number being a property of discrete entities – that the representation of number is analogous to that for continuous dimensions (e.g. time: [5,6]). The behavioral distance effect has been replicated with different input notations (e.g., number words, Arabic numerals) and with dot patterns [7], with a larger number range [8,9], and in both children (e. g. [10-12]) and animals (e.g., [13,14]).

If numerical comparisons are performed on the same abstract notation-independent representation, we should expect common neural correlates of the numerical distance effect regardless of notation. A large body of previous research implicates the intraparietal sulcus (IPS) as an important region for numerical cognition and suggests that this region represents number regardless of whether the input notation is symbolic (e.g., number words or symbols) or non-symbolic (e.g., dot patterns) and regardless of whether stimuli are presented visually or auditorily. For example, using functional magnetic resonance imaging (fMRI), Eger et al. [15] showed that the IPS is activated bilaterally in a numerical target detection paradigm for both visual and auditory numerical arrays. Importantly, the IPS was not activated by color or letter target detection in these two modalities, providing evidence that this activation was specific for the numerical aspect of the task. Using fMRI and event-related potentials (ERPs), Pinel et al. [16] required participants to perform a numerical comparison task ("smaller or greater than 65?") with both visually presented number words and Arabic digits. They found converging evidence from both methods that smaller numerical distances elicited greater parietal brain activity bilaterally irrespective of the input format. These findings suggest that numerical semantic processing relies on a common representation that is independent of the initial input notation and activates the same parietal network.

In another fMRI study, Piazza et al. [17] examined the neural correlates of the distance effect for non-symbolic numerical arrays. They used a passive numerical oddball paradigm (i.e., no task) and presented arrays of shapes of a particular numerosity at a high probability (standard), with an occasional disparate numerosity at a low probability (deviant). In an orthogonal manipulation, on a subset of trials the shape of the elements was varied to allow a test of whether brain regions that responded to shape and number deviation were distinct. They found that the bilateral IPS responded selectively to number changes and not to shape changes. Most importantly, the size of the hemodynamic response was dependent on the difference between standard and deviant numerosity. These results suggest that the IPS, known to be important for symbolic numerical judgments, is also implicated in numerical judgments of non-symbolic numerosities. In a more recent study, Castelli et al. [18] compared brain activity to discrete numerosities and continuous, non-numerical quantities in an estimation task using fMRI. They were able to show that again the bilateral IPS was significantly more active for the discrete numerosity condition than for the continuous, non-numerical quantity condition lending further support to the idea that the IPS is significantly involved in numerical processing, but not processing of non-numerical quantity. And lastly, the IPS also seems to be involved in exact symbolic and approximate non-symbolic addition [19], suggesting notation-independent arithmetic processing in this region.

Although fMRI is quite effective for spatial localization of task-related brain activity, ERPs provide much greater temporal resolution and may therefore provide a complementary window into the underlying processes that cause the numerical distance effect. For instance, it is conceivable that the same brain regions are activated when numerical values are compared in different formats (e.g., numerosities vs. symbols) but that the temporal pattern of this activation differs. In this case, different cognitive processes and mechanisms may ultimately lead to similar hemodynamic brain activations.

Two studies have reported ERP correlates to the numerical distance effect. Both Dehaene [20] and Temple and Posner [12] required participants to judge whether a numerical value was larger or smaller than five. Dehaene [20] tested adults and compared two symbolic conditions – a condition with Arabic numerals and a condition with written number words. Numerical values near to 5 (i.e., 4 and 6) compared to numerical values far from 5 (i.e., 1 and 9) elicited a greater right-lateralized parieto-occipito-temporal positivity that started around 175 ms after stimulus onset for Arabic numerals and 190 ms after stimulus onset for number words. The similarity in topography and time course was taken as support for the hypothesis that different input formats (words and numerals) are translated into a common abstract mental representation and that regardless of input notation the comparison is based on abstract numerical representations.

In contrast, Temple and Posner [12] additionally compared a symbolic and a non-symbolic condition, in this case an Arabic numeral condition and a dot pattern numerosity condition, and tested both adults and 5-year-old children (again the values 1, 4, 6 and 9 compared to a standard of 5). Their results indicated little difference between children and adults in the ERP effects, suggesting that the neural circuits adults use to compare numerosities and symbols are in place by 5 years of age. Results were less conclusive, however, about the parallels between the non-symbolic and symbolic conditions. Results for the Arabic numeral condition largely replicated Dehaene's findings. However, Temple and Posner found a reversal in the polarity of the ERP distance effect for the non-symbolic condition relative to the symbolic condition. In the non-symbolic condition, the parieto-occipito-temporal positivity was greater for the far rather than the near values. Temple and Posner noted this reversal in polarity between the two conditions and speculated that it may have been caused by sensory processing differences between the two conditions but did not explore this hypothesis further. At face value, this reversal in polarity in the ERPs between different input notations contradicts the notion of an input-independent mechanism underlying symbolic and non-symbolic numerical comparisons suggested by fMRI data [16,17]. One possibility is that processing of symbolic and non-symbolic input formats activates the same brain regions with a different temporal pattern that is not apparent in fMRI data.

The goal of our research was to test whether a common cognitive process underlies symbolic and non-symbolic numerical comparisons. If the brain regions recruited by numerical comparison as revealed by fMRI, and the time course of brain activity underlying numerical comparison as revealed by ERPs, are similar for different notational formats, this would provide strong evidence for the idea that a single cognitive process underlies numerical comparison regardless of notational format. Consequently, we examined the temporal and spatial pattern of brain activity during numerical comparison of both symbolic and non-symbolic stimuli to investigate the reason for the reversal in polarity in the ERP distance effect for dots and Arabic numerals found by Temple and Posner [12].

## Experiment 1

The goal of Experiment 1 was to replicate Temple and Posner's experiment and endeavor to examine the cause of the reversal in polarity of the P2p distance effect in the non-symbolic condition compared to the symbolic condition. One important difference between our experiment and previous studies was that we carefully controlled for the total surface area and individual dot size in the non-symbolic condition such that decisions could not be based on overall surface area, individual element size or brightness. Secondly, we used random dot arrays instead of fixed patterns for each numerosity to ensure that participants have to compare the display online. A fixed pattern could be interpreted as another kind of symbol for a numerosity and might not require an online numerical comparison process, especially after some practice. And lastly, we included all values between 1 and 4 and between 6 and 9 inclusive (compared to our comparison standard of 5) to test more than two numerical distances.

### Method

#### Participants

Twelve participants (mean age = 22 ys, 8 female) were recruited from the Duke University community and were either reimbursed for their participation or received course credit. Data from two additional participants were discarded because they completed less than 50% artifact-free trials. All participants gave informed written consent according to the rules of the Duke Institutional Review Board.

#### Task

Participants were presented with Arabic numerals or random dot arrays for the values 1–4 and 6–9 and had to indicate whether the stimulus represented a value smaller or larger than 5. The Arabic numerals (DIGITS) and random dot arrays (DOTS) were presented in separate160- trial blocks, with the test values presented in random order within a block. The Presentation software package (Neurobehavioral Systems, Inc.) was used to control stimulus presentation and to record responses. Participants responded with two fingers of the right hand on a button box (left button for smaller and right button for larger numbers).

#### Stimuli

In the DOTS condition, the random arrangement of black dots on white background subtended a visual angle of approximately 8.6° × 6.5° (400 pixel in width × 300 pixel in height) when seen on a 17" computer screen (36.5 cm × 27.5 cm) from a distance of 60 cm. In AREA DOT trials, the summed area of the dots was constant (A = 10 cm^2^), with the individual dots ranging in size (from radius r = 1.78 cm to r = 0.59 cm). In SIZE DOT trials, the size of the dots was held constant at r = 0.80 cm, and thus the summed area across all dots increased with number. Both conditions were randomly intermixed in each run in the DOTS condition. Additionally, a central fixation cross was displayed throughout the run and participants were asked to fixate on this cross to avoid eye movements.

In the DIGITS condition, Arabic numerals were presented in a bold black Arial 72-point font on white background and subtended a visual angle of approximately 0.57° × 0.81° from a distance of 60 cm.

**Figure 1:**
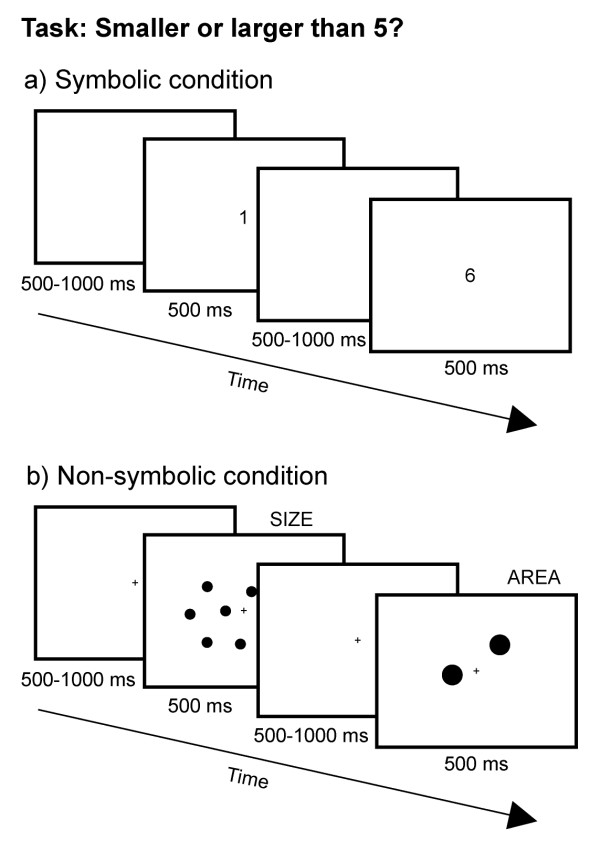
**Experimental design for Experiment 1**. In separate runs, participants were shown Arabic numerals (symbolic condition) or random dot arrays (non-symbolic condition) for the values 1–4 and 6–9 and had to indicate whether the stimulus presented was smaller or larger than 5. Non-symbolic stimuli were controlled for cumulative surface area (AREA) and individual element size (SIZE).

In both conditions, stimulus duration was 500 ms, and trials were separated by a randomly jittered intertrial interval (ITI) of 500 – 1000 ms (see figure 1).

#### Procedure

Ten of the twelve participants completed a total of ten runs, five in each of the two conditions. The other two participants completed a total of twelve runs, four in each of the conditions described above and four in another condition not relevant for the present paper. Each run consisted of 160 trials and lasted about 3 min 20 s. The two conditions were counterbalanced for order. Participants had the opportunity to take breaks between runs. The whole experiment lasted about 40 min of runtime (i.e., not including breaks between runs).

#### Electrophysiological recordings

Brain activity was recorded from 64 electrodes in a custom-made elastic Electrocap (Electrocap, Inc., Eaton, Ohio). The 64 channels included four eye electrodes in order to help detect eye blinks and thereby aid in artifact rejection. Impedances were maintained under 2 k for the mastoid and ground electrodes, under 10 k for the eye-channels, and under 5 k for all the other electrodes. Electrodes were referenced to the right mastoid during recording and later algebraically re-referenced to an average of the right and left mastoids. The electroencephalogram (EEG) was amplified with a gain of 1000. A recording bandpass of 0.01–100 Hz was used, and the EEG was digitized continuously at a rate of 500 Hz/channel onto disk. The recorded EEG was processed off-line with computer algorithms to reject those trials with eye movements, blinks, motion, or other artifacts at any of the channels. The average artifact rejection rate was around 18% (range = 3% – 44%). The data were selectively averaged according to distance and size in each of the two conditions for each individual. Data were normalized using a standardization pulse generated by the system and low-pass filtered with a running nine-point average that attenuated signals at and above 56 Hz (at our digitization rate of 500 Hz). Finally, the mean response time and standard deviation for each subject in each of the two conditions (DIGITS and DOTS) was calculated and only those correct trials that had a RT within the range of one standard deviation above or below the mean for that subject were included to eliminate outlying responses with RTs that were either extremely fast or slow. These procedures yielded an average of 1240 trials per subject, with around 78 trials for each numerosity in each of the two conditions (DOTS and DIGITS) for each subject.

## Results

### Behavioral results

**Figure 2:**
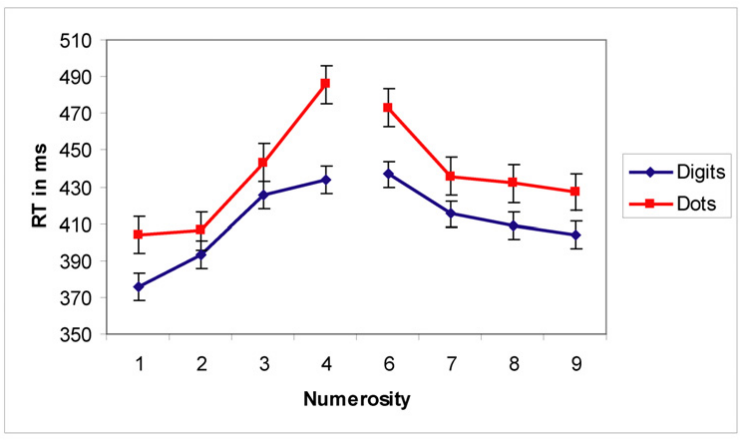
**Behavioral results for Experiment1**. Behavioral results for Experiment 1 show increasing response times for decreasing numerical distance in both conditions (DIGITS and DOTS) and for both small (<5) and large (>5) numerical values. Error bars reflect standard error.

Response times (RTs) displayed the classic distance effect that has been reported in the literature. Mean RTs decreased with increasing numerical distance for both the DIGITS and the DOTS condition (see figure 2). A two-way ANOVA with condition (DIGITS and DOTS) and distance (all four distances) as factors revealed that participants were significantly faster to respond in the DIGITS condition than in the DOTS condition (F(1,11) = 29.40; p < 0.001). A significant main effect of distance (F(3,33) = 104.27, p < 0.001) and a significant interaction between condition and distance (F(3,33) = 9.93, p < 0.001) were also found. The interaction was due to a larger decrease in RT with increasing distance in the DOTS condition. Separate one-way ANOVAs for the DIGITS and DOTS conditions revealed a main effect of distance for each condition (DOTS: F(3,33) = 94.53; p < 0.001; DIGITS: F(3,33) = 41.61; p < 0.001). For the DOTS condition, a two-way ANOVA with trial type (AREA DOTS vs SIZE DOTS) and distance as factors revealed no effect of trial type (F(1,11)<0.01, p = 0.99). Participants responded correctly on an average of 96.6% of all trials (range = 93.75 % – 99.2%). Only correct trials were included in the reported analyses.

### Electrophysiological results

**Figure 3:**
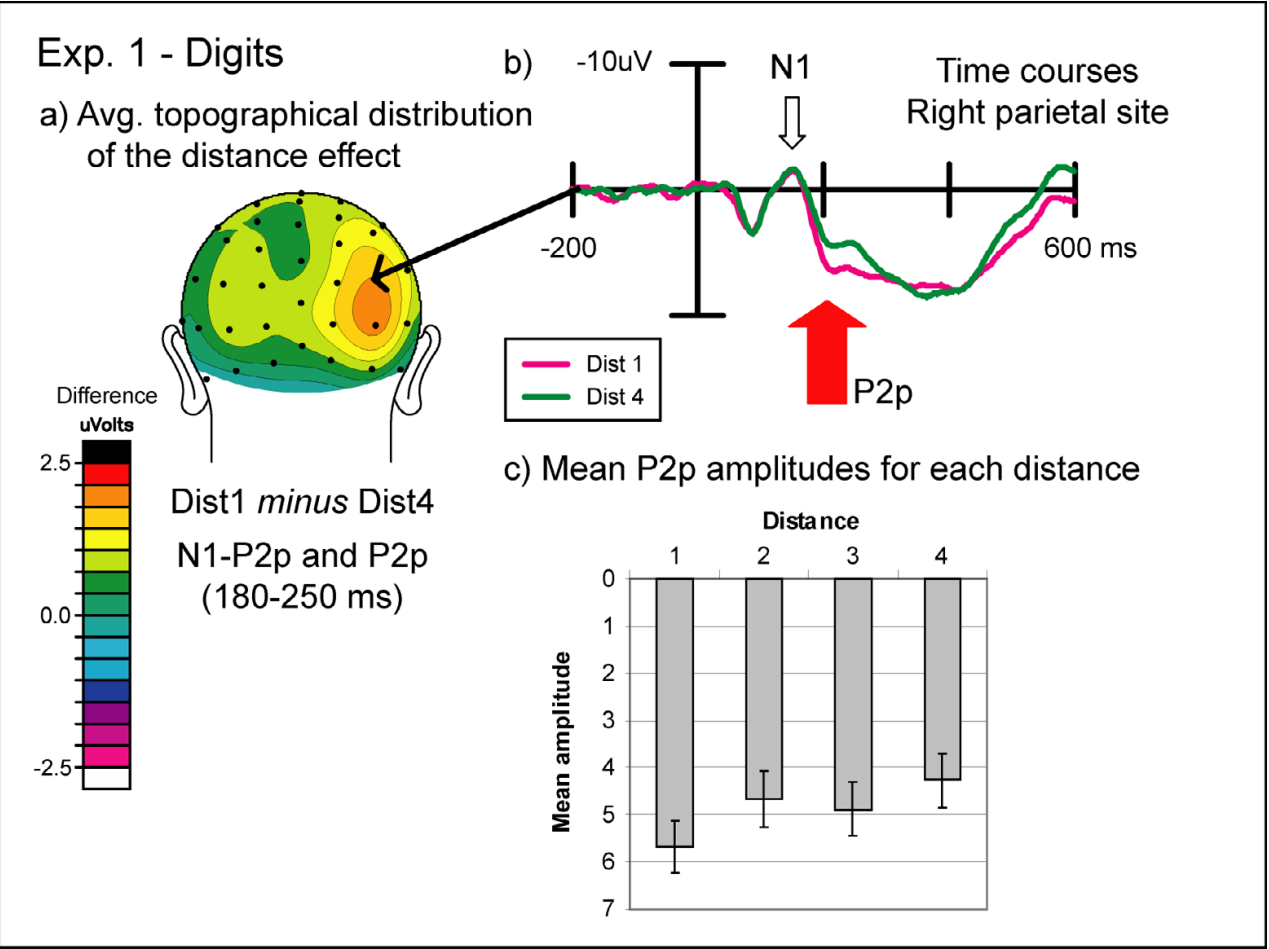
**Experiment 1: symbolic condition**. (a) Topographic distribution of the average brain activity in the symbolic condition of Experiment 1. ERPs to numerals far from 5 (1 and 9) were subtracted from ERPs to numerals near to 5 (4 and 6). There was a strong right lateralized positivity over the N1-P2p transition and the P2p time window. (b) Event-related potentials to symbolic stimuli in Experiment 1. Near and far distances over the right inferior parietal electrode show significant effects of distance between 180 and 250 ms post-stimulus. (c) Mean amplitudes during the P2p time window (210–250 ms) for all four distances. The P2p amplitude decreased with increasing distance.

**Figure 4:**
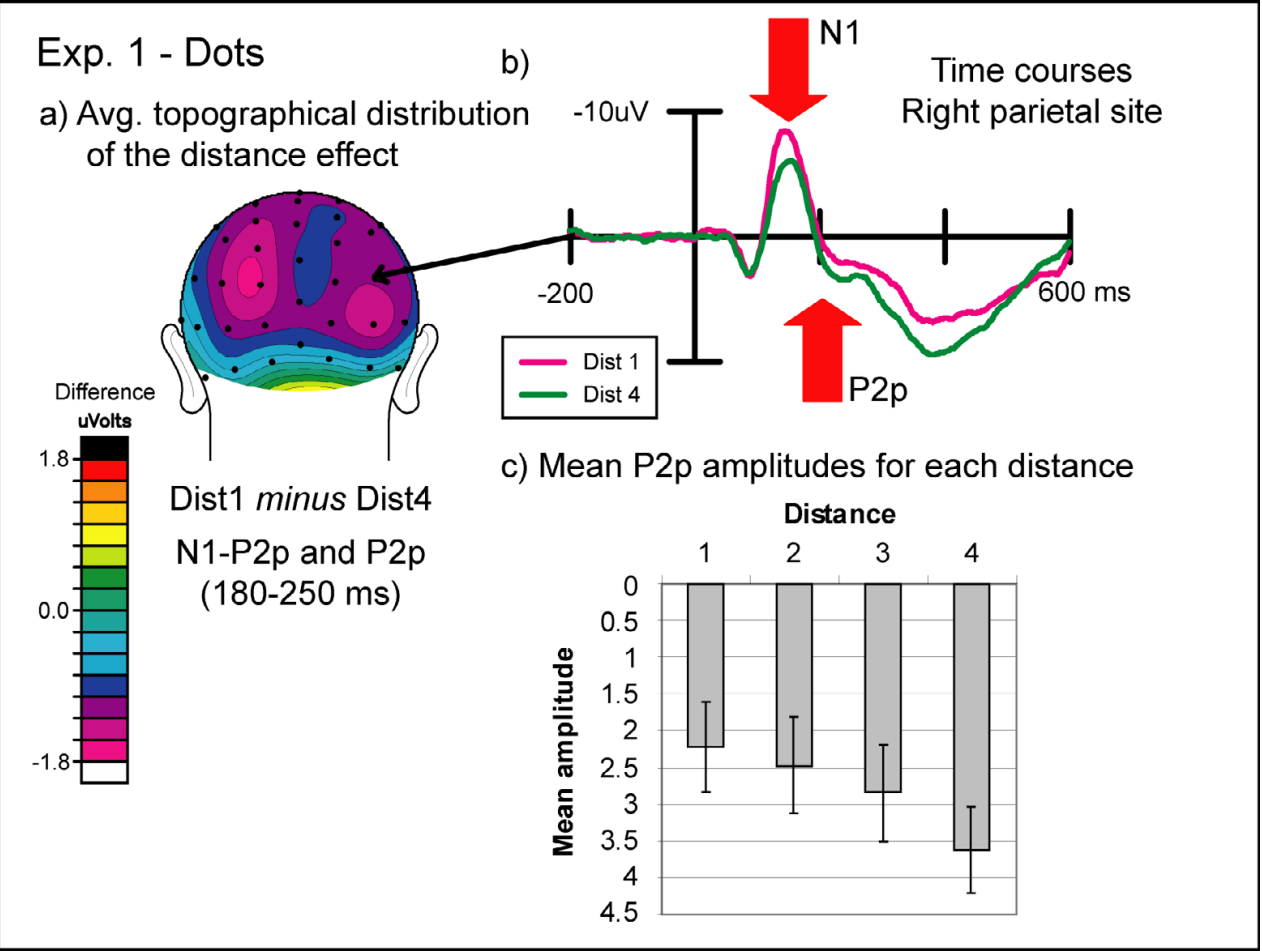
**Experiment 1: non-symbolic condition**. (a) Topographic distribution of the average brain activity in the non-symbolic condition of Experiment 2. ERPs to dot arrays far from 5 (1 and 9) were subtracted from ERPs to dot arrays near to 5 (4 and 6). There was a strong bilateral negativity over the N1-P2p transition and the P2p time window. (b) Event-related potentials to non-symbolic stimuli in Experiment 1. Near and far distances over the right inferior parietal electrode show significant effects of distance starting at 140 ms post-stimulus. (c) Mean amplitudes during the P2p time window (210–250 ms) for all four distances. The P2p amplitude increased with increasing distance.

An initial posterior scalp-positive wave (P1) peaked at around 90 ms after stimulus onset for DOTS and around 120 ms for DIGITS. Since previous reports did not lead us to anticipate any effects from the present task on this early visual sensory component, we did not analyze it any further. The P1 was followed by a posterior negativity (N1) peaking at around 155 ms post-stimulus in both the DIGITS and DOTS conditions (see figures 3 and 4). The N1 was followed by a second posterior positivity (P2p) that peaked around 230 ms post-stimulus. Finally, a broadly distributed late positivity (P3), largest over central and parietal leads, was observed peaking on average at 405 ms after stimulus onset in the DIGITS condition and 440 ms in the DOTS condition, which was around the time the mean behavioral response was made. Peak latencies for different ERP components were determined based on the average of six parietal electrodes (three over each hemisphere) for the P1, N1, and P2p components and the average of four central electrodes (two over each hemisphere) for the P3 component.

Four non-overlapping time windows centered on these peaks were chosen: N1: 138–172, N1-P2p: 180–202, P2p: 210–250, P3: DIGITS: 355–455, DOTS: 390–490. Since the P3 is often substantially influenced by manual responses and response times (e.g. [21,22]), and RT differed in the two conditions, it was necessary to use different time windows for the P3 for the DIGITS and DOTS condition. The windows reported here are very similar to the time windows used by Dehaene [20] and Temple and Posner [12]. We analyzed the effect of distance and size on a subset of electrode sites chosen based on proximity to sites reported in previous studies (temporo-occipital [TO] and inferior parietal [IP] electrode pairs) for the N1, N1-P2p, and P2p components, and on an average over two central electrode pairs for the P3 component. Temporo-occipital scalp sites were located between O1 and T5 (left hemisphere) and between O2 and T6 (right hemisphere) in the standard 10–20 system, inferior parietal scalp sites were inferior to P3 (left) and P4 (right), and central scalp sites were posterior to C1 and close to C3 (left hemisphere) and posterior to C2 and close to C4 (right hemisphere). All ANOVAs were performed on the mean amplitudes over the test windows and included as factors all four distances (distance of 1, i.e. the numerical values 4 and 6, distance of 2, i.e. 3 and 7, distance of 3, i.e. 2 and 8, and distance of 4, i.e. the numerical values 1 and 9), size (small vs. large numerical values) and hemisphere (left vs. right), unless otherwise noted. The p-values were Greenhouse-Geisser corrected (pGG) when the assumption of sphericity was violated (ε<0.75).

#### N1 component

**Figure 5:**
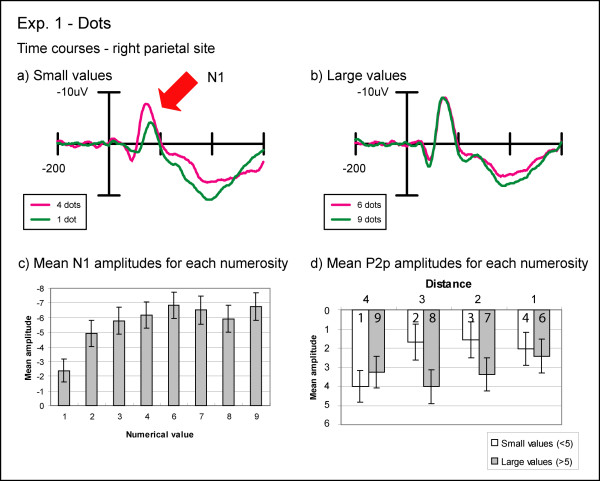
**Experiment 1: ERPs to non-symbolic stimuli separated by numerical value**. (a & b) These figures depict brain responses to the numerosities 1, 4, 6, and 9 used in the dot displays in Experiment 1 for the right inferior parietal electrodes. These data strongly suggest that the N1 effect observed in figure 4 is due to differences in the N1 component for small numerical values. (c) The mean amplitudes during the N1 time window decrease significantly with increasing numerical value between 1 and 6. (d) In this figure, the mean amplitudes for each numerosity during the P2p time window are grouped by distance. Small numerical values show a significantly smaller P2p amplitude than large numerical values when distance is held constant.

In the DIGITS condition, we found a significant effect of distance on the amplitude of the N1 component over both inferior parietal and temporo-occipital sites (IP: F(3,33) = 3.56, p = 0.02; TO: F(3,33) = 10.31, pGG<0.001). This effect was mainly caused by a larger N1 amplitude to the digit seven than the other digits as evidenced by an additional significant interaction between distance and numerical size (IP: F(3,33) = 3.94, pGG = 0.03; TO: F(3,33) = 4.80, p < 0.01). No other significant effects on the N1 were found in the DIGITS condition. In the DOTS condition, a significant main effect of distance was found at both sites (IP: F(3,33) = 15.91, p < 0.001; TO: F(3,33) = 49.55, p < 0.001), the effect being due to a greater N1 amplitude for small distances compared to large distances. Furthermore, there was a significant main effect of numerical size over both sites (IP: F(1,11) = 20.41, p < 0.001; TO: F(1,11) = 27.88, p < 0.001), which was due to a smaller N1 amplitude for small values than large values and a significant interaction between distance and size (IP: F(3,33) = 14.99, pGG<0.001; TO: F(3,33) = 34.58, pGG<0.001). The interaction appeared to reflect a more pronounced effect of distance for small compared to large values (see figures 4 and 5). Lastly, a significant main effect of hemisphere was observed over the inferior parietal electrode pair (F(1,11) = 7.04, p = 0.02), which appeared to be due to a greater N1 amplitude over the right than over the left hemisphere.

#### Transition from N1 to P2p

In the DIGITS condition, the transition between the N1 and the P2p components showed a significant main effect of distance over both pairs of sites (IP: F(3,33) = 4.63, pGG = 0.02; TO: F(3,33) = 13.98, p < 0.001), with numbers near to the standard value eliciting a greater ***positivity ***than those further away from the standard (see figure 3). In the DOTS condition, there was a significant main effect of distance over both scalp locations (IP: F(3,33) = 10.69, p < 0.001; TO: F(3,33) = 9.02, pGG<0.01), but examination of the data pattern revealed that the amplitudes for values near the standard were more ***negative ***than for values further away from the standard (see figure 4). In addition, the DOTS condition showed a significant effect of numerical size (IP: F(1,11) = 5.44, p = 0.04; TO: F(1,11) = 9.01, p = 0.01) and a significant interaction between distance and size over both pairs of electrode locations (IP: F(3,33) = 7.23, p < 0.01; TO: F(3,33) = 7.44, pGG<0.01). The interaction was again due to a more pronounced effect of distance for small compared to large values.

#### P2p component

For the DIGITS condition in the P2p time window, a significant distance effect was found for both sites (IP: F(3,33) = 5.23, pGG = 0.01; TO: F(3,33) = 6.15, p < 0.01). Small distances from the standard exhibited a greater positivity than large distances (see figure 3). In addition, this distance effect was significantly more pronounced over the right than over the left hemisphere as evidenced by a significant interaction between distance and hemisphere (IP: F(3,33) = 4.49, pGG = 0.02; TO: F(3,33) = 3.47, pGG = 0.05). In the DOTS condition, we found again a significant main effect of distance over both sites (IP: F(3,33) = 6.81, pGG<0.01; TO: F(3,33) = 6.65, p < 0.01), with small distances being less positive than large distances (see figure 4). Furthermore, small numerical values exhibited a significantly smaller P2p amplitude than large numerical values over both sites (IP: F(1,11) = 10.80, p < 0.01; TO: F(1,11) = 7.83, p = 0.02), and the distance effect was significantly more pronounced for small than for large numerical values as evidenced by a significant interaction between distance and size (IP: F(3,33) = 9.08, p < 0.001; TO: F(3,33) = 9.06, pGG<0.01).

#### P3 component

The P3 component for the DIGITS condition did not show a main effect of distance. There was a significant effect of hemisphere with the amplitude of the P3 being significantly greater over the right than over the left central scalp (F(1,11) = 6.82, p = 0.02). Additionally, there was a significant interaction between size and hemisphere (F(1,11) = 25.56, p < 0.001). This effect was apparently due to a larger size effect over the left hemisphere than over the right. There were no significant differences in peak latency for the four distances in the DIGITS condition.

For the P3 component in the DOTS condition, we found a significant effect of distance over the central electrode sites (F(3,33) = 9.04, pGG<0.01), which was caused by a smaller positive amplitude for the small distances. There was also a significant main effect of size (F(1,11) = 14.76, p < 0.01) that was due to a greater positivity for small values as compared to large values. Furthermore, there was a significant main effect of hemisphere (F(1,11) = 13.38, p < 0.01) and a significant interaction between size and hemisphere (F(1,11) = 14.45, p < 0.01). These effects appeared to be due to a greater P3 amplitude over the right hemisphere and larger size effects over the left hemisphere. Furthermore, there was a significant difference in the peak latency of the P3 for the four distances (F(3,33) = 4.57, pGG = 0.02). Thus, we also compared the mean P3 amplitudes for different time windows for each distance centered around the respective peaks (dist1: 415–515 ms, dist2: 387–487 ms, dist3: 386–486 ms, dist4: 363–463 ms). The pattern of results was the same as for the fixed time window reported above, except for an additional marginally significant interaction between distance and size (F(3,33) = 2.89, p = 0.05) that seemed to be due to a larger distance effect for the large than the small numerical values.

#### Sensory control in the DOTS condition

There were no effects of dot trial type (AREA DOT vs SIZE DOT) on any of the time windows for the DOTS condition.

## Discussion

Our electrophysiological results show clear distance-related changes in brain activity over temporo-occipital and parietal electrode sites starting around 180 ms post-stimulus. The second posterior positivity (P2p) component showed a greater positivity for Arabic numerals closer to our comparison numerical standard of five as compared to those further away, replicating previous findings [12,20]. This effect was larger over the right than over the left hemisphere, which is also in line with previous findings [20]. These electrophysiological effects parallel our behavioral measures, which show the classic reaction time (RT) distance effect of slower RTs for numerically closer distances.

A quite different pattern of results was obtained for non-symbolic DOTS stimuli. Similar to the results reported by Temple and Posner [12], the N1 component showed a significant effect of distance that was absent in the symbolic condition, with the ERPs being more negative in the N1 time window for dot patterns at small distances. In addition, as in Temple and Posner, the ERPs for the small distances remained more negative during the transition to the P2p, thus showing the opposite increased positivity pattern elicited by the Arabic numerals. However, examining the ERPs separately as a function of numerical size revealed that the amplitude of the N1 component for the DOTS condition increased dramatically with increasing numerical value for values less than 5 and changed only slightly for values larger than 5 (see figure 5c). Therefore, the resulting distance effect in the N1 for the DOTS trials was not driven by numerical distance per se but rather by differences in the absolute numerical values as indicated by a significant size effect and a significant interaction between distance and size. Importantly, this N1 size effect and potentially other effects due to differences in sensory processing seem to extend into the P2p time window. As can be seen in figure 5d, there are still significant differences in the P2p amplitude when the small and large numerical values for each distance are compared. Here, small numerical values elicit smaller P2p amplitudes than large numerical values even if their numerical distance to the standard is the same. We attribute these differences to differences in sensory processing for the DOTS conditions, especially for the small numerical values that initially start during the N1 time window but extend into the P2p time window and interact with the numerical distance effect. Even for large numerical values, the N1 amplitudes vary slightly making it difficult to clearly differentiate between the effects of sensory and numerical comparison processes on the ERPs.

At first glance, these findings suggest a distinction between small and large number processing observed in different kinds of numerical tasks (e.g. adult subitization effects or infant set size limitations). However, it seems unlikely that the size effect observed in our data is due to distinct enumeration processes for small and large values. First, the behavioral data shows clear symmetrical distance effects for both small and large values (see figure 2) and, if anything, a greater increase in RT for decreasing distance for small values. Furthermore, it is important to note that RT in this task reflects a multipart process of forming numerical representations *and *making numerical comparisons. Secondly, the amplitude of the N1 increases smoothly between the values of 1 and 6 (see figure 5c), which is already beyond the typical subitizing range [23], making a distinct enumeration process between small and large values highly unlikely. Thus, it seems more likely that the amplitude of the N1 is more pronounced for small than for large numbers due to differences in sensory processing. The visual N1 ERP component seems to be modulated by a variety of sensory stimulus aspects. Differences in the N1 amplitude were found in studies in which the luminance of the visual stimuli changed [24], with stimuli eliciting illusory contours [25] and in studies varying the visual perceptual load [26]. The N1 component has also been found to reflect differences in sensory processing in other numerical tasks (e.g. [20,27]). Lastly, it is known that relatively subtle stimulus differences can result in confounding effects on sensory ERP components [28]. Thus, it is conceivable that differences in cumulative contour length (i.e., differences in the amount of edges) or perceptual load between small and large non-symbolic stimuli might have caused the N1 effects observed here.

In addition to the effects during the N1 and the P2p time windows, we observed differences in the distance effect on the P3 amplitude between the symbolic and the non-symbolic conditions. There was no significant distance effect on the P3 amplitude in the symbolic condition, nor differences in peak latency of the P3. In contrast, the peak latency of the P3 increased with decreasing distance from the standard in the non-symbolic condition. We observed a clear distance effect on the P3 amplitude that was present even when the time window of interest was adjusted to center around the respective P3 peaks of each distance. This discrepancy between the symbolic and the non-symbolic condition could be due to the stable level of response confidence for all distances in the well-known symbolic format as compared to the less well-known non-symbolic format in which participants might have been less confident especially in their responses to small distances [20].

In summary, consistent with previous reports, we found that both the N1-P2p transition and the P2p component itself were correlated with numerical distance in both a symbolic numeral condition and a non-symbolic numerosity condition. However, also consistent with previous reports, we found that in the symbolic condition the parieto-occipito-temporal positivity was greater for the small distances, whereas in the non-symbolic condition the polarity was reversed with the parieto-occipito-temporal positivity being greater for the large rather than the small distances. This difference in polarity would argue against the view that the N1-P2p transition and the P2p component are general neural correlates of numerical distance irrespective of numerical notation. However, the asymmetrical effect of distance in the non-symbolic condition for values smaller and larger than the standard would lead us to hypothesize that differences between the symbolic and non-symbolic condition were caused by differences in sensory processes for small arrays in the latter condition. For example, it may have been the case that the enhanced scalp negativity resulting from a substantially larger N1 amplitude for arrays with larger versus smaller numerical values contaminated the immediately following ERP activity in the N1-P2p transition and P2p latency ranges and resulted in a reversed polarity "distance" effect (i.e., less positivity) for near-to-five distances than for far-from-five distances.

## Experiment 2

The results of Experiment 1 suggested that the reversal in polarity of the distance effect in the non-symbolic condition relative to the symbolic condition might be due to confounding sensory ERP differences for the small-values dot sets. To test this hypothesis, a second experiment was carried out in which small numerical values were eliminated. Participants were presented with dot arrays that contained between 8 and 30 elements and were required to indicate whether the value presented was smaller or larger than 15. Additionally, we controlled for cumulative contour length of the stimuli to minimize potential edge effects on the N1 and other sensory ERP components.

### Method

Experiment 2 differed from Experiment 1 only in the following aspects:

#### Participants

Fourteen participants (mean age = 24 years, 8 female) were recruited from the Duke University community and were either reimbursed for their participation or received course credit. Data from 6 additional participants were discarded because they completed less than 50% artifact-free trials (N = 3) or they responded incorrectly on more than 25% of their trials (N = 3).

#### Task

Participants were presented with random dot arrays and had to indicate whether the array represented a numerosity smaller or larger than 15. On one third of the trials, the size of the dots was constant across numerosities (SIZE DOTS), on another third, total surface area was constant across numerosities (AREA DOTS) and on the final third, total perimeter was constant across numerosities (PERI DOTS).

#### Stimuli

Discrimination of large numerosities typically follows Weber's law; thus, here we used target values that differed equally in ratio from the standard (standard = 15 and test values = 8, 10, 11, 12, 19, 20, 23, and 30). The values 12 and 19 were considered "distance 1", the values 11 and 20 were labeled as "distance 2", the values 10 and 23 were labeled as "distance 3", and the values 8 and 30 were considered "distance 4." The random arrangement of black dots on a white background subtended a visual angle of approximately 10.75° × 6.5° (500 pixel in width × 300 pixel in height) when seen on a 17" computer screen (36.5 cm × 27.5 cm) from a distance of 60 cm. In SIZE DOTS trials, each circle had a radius of 0.6 cm yielding cumulative surface areas between 9.04 cm^2 ^and 33.90 cm^2 ^and cumulative perimeters between 30.16 cm and 113.10 cm. In AREA DOTS trials, the summed area of the dots was constant (A = 15 cm^2^). Thus, the dots changed in size from radius r = 0.77 cm to r = 0.40 cm and in cumulative perimeter from p = 38.96 cm to p = 75 cm. In PERI DOTS trials, the cumulative perimeter of the dots remained constant (p = 60 cm), thus, the radius and summed area across all dots increased with number (radius range: 1.19 cm – 0.32 cm; area range: 9.6 cm – 35.92 cm). All three trial types were randomly intermixed in each run. Additionally, a central fixation cross was displayed throughout the run and participants were asked to fixate on this cross to avoid eye movements.

As in Experiment 1, each stimulus was presented for 500 ms and trials were separated by a randomly jittered intertrial interval (ITI) of 500 – 1000 ms.

#### Procedure

Each participant completed a total of eight runs. Each run consisted of 160 trials and lasted about 3 min 20 s. Participants had the opportunity to take breaks between runs. The experiment contained 27 min of total runtime. Before the first run, each participant was familiarized with the task and shown three examples of 15 dots, and three examples of each of the numerosities used in the experiment.

#### Electrophysiological recordings

Electrophysiological recordings were the same as in Experiment 1.

## Results

### Behavioral results

**Figure 6:**
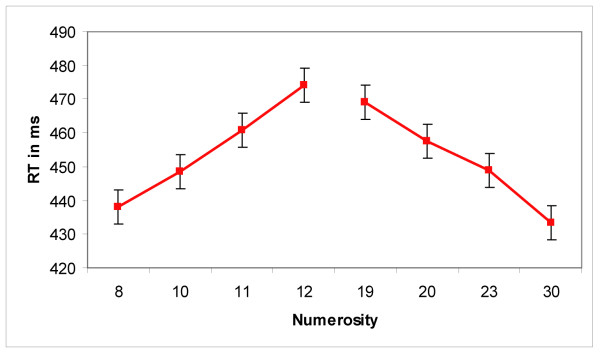
**Behavioral results for Experiment 2**. Behavioral results for Experiment 2 show increasing response times for decreasing distance to the standard value of 15. The symmetry of the RT function shows that response time is modulated by the ratio between the numerosities. Error bars reflect standard error.

RTs displayed the usual distance effect reported in the literature. A two-way ANOVA with trial type (AREA DOTS, PERI DOTS, SIZE DOTS) and distance (all 4 distances) as factors revealed a main effect of distance (see figure 6; F(3,36) = 3.468, p = 0.026). There was also a main effect of trial type (F(2,26) = 4.355, p = 0.02). Pairwise t-tests showed that there were no significant differences between the AREA DOTS and SIZE DOTS trials (t(13) = 0.55, p = 0.59), but participants were significantly slower on PERI DOTS trials compared to SIZE DOTS trials (t(13) = 2.79, p = 0.02) and AREA DOTS trials (t(13) = 2.26, p = 0.04). A between-group comparison revealed no significant differences between the RTs in the DOTS conditions of Experiments 1 and 2 (t(24) = 0.76, p = 0.46). Only correct trials are included in the following analysis (on average 90.9% of all trials).

### Electrophysiological results

**Figure 7:**
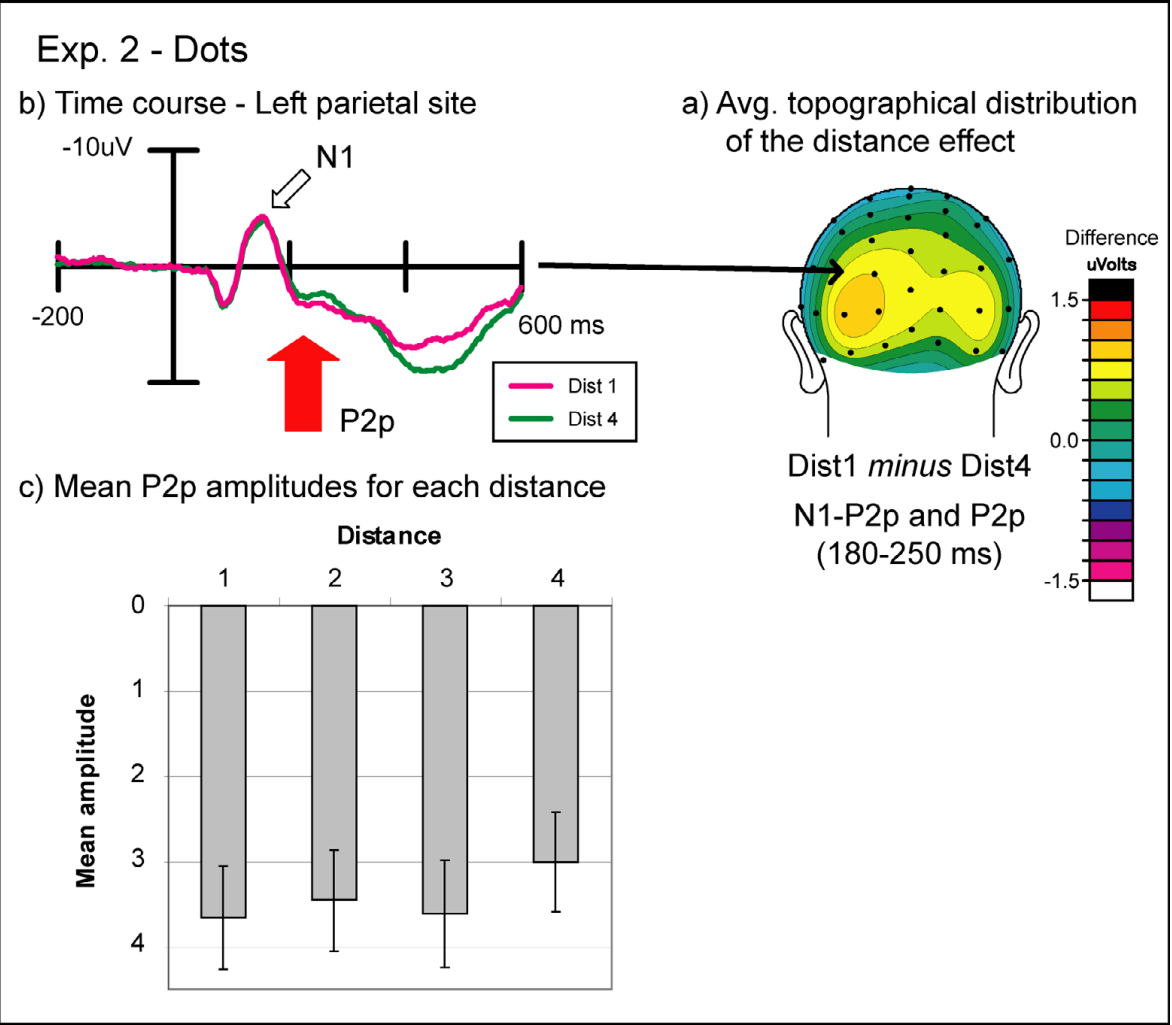
**Experiment 2: non-symbolic condition**. (a) Topographic distribution of the average brain activity in the non-symbolic condition of Experiment 2. ERPs to numerals far from 15 (8 and 30) were subtracted from ERPs to numerals near to 15 (12 and 19). There was a strong bilateral positivity over the N1-P2p transition and the P2p time window. (b) Event-related potentials to non-symbolic stimuli in Experiment 2. Near and far distances over the left inferior parietal electrode show significant effects of distance starting at 210 ms post-stimulus. The pattern in brain response here is similar to that observed for Arabic numerals in Experiment 1 (see figure 3). (c) Mean amplitudes during the P2p time window (210–250 ms) for all four distances. The P2p amplitude decreased with increasing distance.

The general wave shapes obtained in Experiment 2 were virtually identical to those obtained in Experiment 1 (see figure 7); and thus accordingly, with the exception of the P3 which peaked somewhat later than in Experiment 1, the same time windows were used. The P3 in Experiment 2 peaked at 490 ms after stimulus onset, again around the time the average behavioral response was made. Thus, we chose a slightly later time window for the P3 here (440–540 ms post-stimulus). Electrode sites were the same as for Experiment 1. Again, p-values were Greenhouse-Geisser corrected when the assumption of sphericity was violated (ε<0.75).

#### N1 component

Here in Exp. 2, no significant effects were found on the N1 component, neither as a function of distance, numerical size, or trial type.

#### Transition from N1 to P2p

In the transition phase between N1 and P2p, there was a significant main effect of distance over temporo-occipital sites (F(3,39) = 4.94, pGG = 0.01) that was due to a greater positive amplitude for small distances compared to large distances. A significant main effect of size was found over both parietal and temporo-occipital sites (IP: F(1,13) = 7.56, p = 0.02; TO: F(1,13) = 8.25, p = 0.01) which appeared to be due to a greater amplitude for large numerical values as compared to small. No significant interaction between distance and size was found. Furthermore, a significant interaction between size and hemisphere was observed over temporo-occipital sites (F(1,13) = 6.46, p = 0.02), which appeared to be due to a greater size effect over the right than over the left hemisphere.

#### P2p component

Both electrode locations showed significant distance effects during the P2p time window (IP: F(3,39) = 3.45, p = 0.02; TO: F(3,39) = 10.05, pGG<0.001). Again, small distances were more positive than large distances. In addition to these distance effects, we found significant effects of size over both the IP and TO sites (IP: F(1,13) = 14.71, p < 0.01; TO: F(1,13) = 8.42, p = 0.01). Similar to the size effect in the N1-P2p transition, small numbers were less positive than large numbers. However, there was no significant interaction between distance and size at either of the two sites.

#### P3 component

The P3 component showed a significant effect of distance (F(3,39) = 24.11, pGG<0.001) that appeared to be due to large distances being more positive than small distances over central sites. Furthermore, a significant interaction between size and hemisphere was found (F(1,13) = 16.14, p < 0.01) that appeared to be due to a greater size effect over the left hemisphere. There were no significant differences in peak latency for the four distances, so no further analysis of the P3 (e.g., within adjusted windows) were warranted.

#### Sensory control

Separate ANOVAs with main effects of trial type (AREA DOTS, SIZE DOTS, PERI DOTS), distance (all four distances) and size (small vs large numerical values) were conducted at each of the four time windows. Significant main effects of trial type were found in all time windows over both temporo-occipital and parietal sites (*N1 *– IP: F(2,26) = 7.53, p < 0.01; *N1-P2p *– IP: F(2,26) = 13.06, p < 0.001; TO: F(2,26) = 5.39, p = 0.01; *P2p *– IP: F(2,26) = 7.46, p < 0.01; TO: F(2,26) = 3.54, p = 0.04; *P3 *– F(2,26) = 20.26, p < 0.001), but no significant interactions between trial type and distance or size were found at any location in any of these time windows. The main effects of trial type appeared to be due to a somewhat smaller N1 amplitude and greater P2p and P3 amplitudes in the PERI condition relative to the AREA and SIZE conditions.

## Discussion

The main finding of Experiment 2 was a significant distance effect over bilateral temporo-occipital and parietal sites in the P2p latency that was of the *same *polarity as the symbolic condition in Experiment 1, with small distances eliciting a larger positivity than large distances. This distance effect started around 180 ms post-stimulus over bilateral temporo-occipital sites and was significant over both temporo-occipital and inferior parietal sites around the peak of the P2p component, i.e. around 210–250 ms after stimulus onset. Importantly, in contrast to our results obtained with non-symbolic stimuli in Exp. 1, we did not find any significant effects on the N1 component or any interaction between distance and numerical size in this experiment. However, we did find a significant effect of numerical distance on the P3 amplitude as was the case in the non-symbolic condition in Exp. 1. As mentioned before, it is possible that this difference between symbolic and non-symbolic conditions may be due to less response confidence in the non-symbolic condition that is greater for the smaller as compared to the larger distances.

## General discussion

There are two main conclusions that can be drawn from the experiments reported here. First, abstract numerical semantic processing as indexed by an electrophysiological numerical distance effect for numerical comparisons is reflected by differences in the transition from the first ERP negativity (N1) to the second posterior positivity (P2p) that starts around 180 ms after stimulus onset and on the P2p component itself. This effect is most pronounced over temporo-occipital and inferior parietal areas. Our results suggest that previously reported earlier-latency sensory effects, especially on the N1 component, may not reflect numerical processing because the distance effects interacted with effects of numerical size and appeared to be due to a sensory confound.

The second conclusion is that the P2p ERP distance effect is common to Arabic numerals and non-symbolic dot arrays, suggesting that it does indeed reflect an abstract and notation-independent neural activation that is invoked for both symbolic and non-symbolic numerical processing. Using numerical values between 1 and 9 (Exp. 1), we replicated differences between the Arabic numerals and the dot pattern condition found in previous reports such that small distances were more positive than large distances for Arabic numerals but less positive for random dot arrays. Closer analysis of this difference, however, revealed that the amplitude of the N1 component increased with increasing numerical value in the non-symbolic condition. Therefore, the resulting distance effect in the N1 was not driven by numerical distance per se but rather by the absolute numerical values. These differences in sensory processing seemed to extend into the later processing stages resulting in the polarity differences in the P2p component. By testing participants with all large numerical values in Exp. 2 (8 – 30), differences in early sensory processing were eliminated, thereby allowing distance-related differences in the P2p component that exhibited the same pattern in polarity as the Arabic numerals in Experiment 1 to be revealed. This distance-related difference in the P2p was more pronounced over the right than the left hemisphere in the symbolic condition, but was more bilateral for the non-symbolic condition, a pattern that has been observed in previous studies [12,20].

These results support the claim of a notation-independent magnitude representation for number as hypothesized in the triple-code model proposed by Dehaene and Changeux [29]. The model suggests three distinct systems underlying numerical processes: a quantity system that includes the abstract semantic representation of numerosities, a verbal system that is mainly concerned with the linguistic aspects of number words, and a visual system that is mainly concerned with the symbolic representation of numbers, such as in the form of Arabic numerals [30]. According to the model, the quantity system is involved whenever semantic information needs to be accessed in a numerical task, as was the case in our experiments. Our results support the idea that semantic numerical information is not stored in distinct notation-dependent formats (i.e. differently for symbolic and non-symbolic notations), but rather in an abstract input-independent format. Neuroimaging studies suggested that a key component of this quantity system may be localized in the bilateral IPS, more specifically in the horizontal segment of the IPS [16-18]. In addition to this spatial overlap in brain activity, our results show that the temporal pattern of brain activity in the numerical comparison stage is quite similar for symbolic and non-symbolic number notations. Thus, these two lines of evidence strongly support the idea of a notation-independent representation of number.

Interestingly, Turconi et al. [27] found that the verbal phrasing of the numerical comparison task yielded differences in the time course and localization of brain activity as reflected in ERP recordings. Specifically, they required participants to determine whether an Arabic numeral "was smaller or larger than 15" (quantity judgment) or instead whether the numeral "came before or after 15" (order judgment). Despite the superficial similarity between the two conditions, the distance effect in the quantity judgment was left-lateralized and started around 170 ms after stimulus onset whereas for order judgments the effect was bilateral and started around 210 ms after stimulus onset. Nevertheless, their findings are consistent with the current results in that the distance effect in the quantity judgment was observed at approximately the same time period and location (albeit left-lateralized) as the P2p distance effect observed in our task.

It is important to note that our experiments did not examine the specificity of the neural substrate for numerical distance effects. In this paradigm, it is impossible to distinguish if the representation is specific to the number domain or if it is also used to represent quantities in general – i.e. continuous quantities such as surface area, weight, or time, or order information. For instance, Turconi et al. [27] found similar effects on the P2p in order judgments on letters, suggesting that the distance-related changes in the P2p component might not reflect number-specific processes.

In conclusion, our results suggest a notation-independent neural process underlying the numerical distance effect that starts around 180 ms after stimulus onset and that is reflected by differences in the transition between the first ERP negativity (N1) and the second posterior positivity (P2p) and that continues across the P2p ERP component. We suggest that differences in the neural correlates of the distance effects for symbolic and non-symbolic numerical stimuli found in previous studies stem from differences in sensory processing. If these confounding sensory effects are eliminated, such as by using exclusively large numerical values as we did here, the commonality of the underlying notation-independent neural processes is revealed.

## Abbreviations

ERP = event-related potential, IPS = intraparietal sulcus, fMRI = functional magnetic resonance imaging, ITI = intertrial interval, EEG = electroencephalogram, RT = response time, P1 = first positivity, N1 = first negativity, P2p = second posterior positivity, P3 = third positivity, IP = inferior parietal, TO = temporo-occipital

## Competing interests

The author(s) declare that they have no competing interests.

## Authors' contributions

MEL, MGW and EMB conceived the experiment. MEL collected the data. MEL and MGW designed and carried out the data analysis. MEL, MGW and EMB wrote the article.
